# Towards the Improvement of Patient Experience Evaluation Items for Patient-Centered Care in Head and Neck Cancer: A Qualitative Comparative Study

**DOI:** 10.3390/healthcare12121164

**Published:** 2024-06-08

**Authors:** Eun-Jeong Kim, Yoo-Ri Koo, Inn-Chul Nam

**Affiliations:** 1 Department of Industry-Academic Cooperation Foundation, The Catholic University of Korea, Seoul 06591, Republic of Korea; dodam.design.research@gmail.com; 2 Department of Service Design, Graduate School of Industrial Arts, Hongik University, Seoul 04066, Republic of Korea; yrkoo@hongik.ac.kr; 3 Department of Otorhinolaryngology-Head and Neck Surgery, Incheon St. Mary’s Hospital, The Catholic University of Korea, Seoul 21431, Republic of Korea

**Keywords:** head and neck cancer, patient experience evaluation, patient needs, patient-centered care

## Abstract

Owing to long-term treatment, frequent consultations, and complications, the evaluation of patients with head and neck cancer (HNC) must be improved. This study explored an opportunity for improving patient experience (PE) evaluation of patients with HNC to achieve a patient-centered, integrative evaluation model based on patient needs. The study comprised four phases: (1) a systematic literature review of PE factors for patient quality of life (QoL) and establishment of PE factor categories as a framework, (2) a review of current cancer or HNC PE evaluation tools, (3) identification of potential PE evaluation items based on patient needs by conducting user research, and (4) suggestion of integrative HNC PE evaluation items through expert validation. As a result, the 39 potential items were initially identified from the literature review and user research. After conducting two survey rounds with experts, 25 items were suggested as HNC PE evaluation items. These underscore the importance of highlighting the patient’s participation, the medical staff’s comprehensive information delivery, empathy, and collaborative communication, the hospital’s support of communication channels, the medical environment for patient emotional support, the education program, and systematic patient satisfaction data management. PE evaluation items that consider the diverse perspectives of stakeholders involved in HNC treatment and factors of comprehensive PE will contribute to improving HNC patient-centered care (PCC).

## 1. Introduction

### 1.1. Study Background

Patient-centered care (PCC) emphasizes identifying patients’ diverse needs, including their preferences, values, and interests, to improve their overall care experience [1,2]. Identifying the needs from a patient’s perspective can improve treatment outcomes and patient satisfaction. Therefore, effective and timely communication with the patient is an essential component of patient-centered perioperative care [1]. PCC includes diverse aspects, from appropriately treating the patient to guiding the patient’s decision making throughout the treatment process and minimizing unnecessary treatment [1].

Patient satisfaction measures used to identify areas of treatment that require improvement are increasingly recognized as key performance indicators for assessing the quality of care to achieve patient-centeredness. Recently, PCC has gone beyond simply identifying patient expectations and praise for treatment. It is evaluated using the patient experience (PE) measure, which assesses the patient’s feelings and experiences while receiving treatment [2].

Cancer treatment depends highly on multidisciplinary medical services, often based on long and frequent patient interactions [3]. Healthcare professionals must identify and address the various needs of the people they care for, from diagnosis to hospitalization or post-discharge home service [4]. Previous studies have shown that cancer patients experience unmet needs during and after treatment [5]. However, conventional measures do not reflect the patient’s perspective, and most items do not provide a current evaluation based on PE [6]. Problems experienced by patients, unmet needs, and stressors affect their satisfaction with care, treatment adherence, and quality of life (QoL) [7]. In particular, psychological and physical aspects should be considered essential for the QoL and well-being of patients in their everyday lives [8,9]. Treatment compliance decreases, treatment is abandoned midway, or poor results are obtained, making it challenging for patients to return to daily life when these aspects are not adequately supported [9,10,11]. Furthermore, social support, such as forming networks outside the family [12], is highly correlated with the health of individuals with chronic diseases [13].

Head and neck cancer (HNC) is characterized by fast-growing tumors in anatomically delicate and functionally fragile areas, which suggests that the sequelae of tumor progression, resection, and nonsurgical management can lead to permanent functional decline [14]. Patients generally receive surgery, radiation therapy, chemotherapy, and targeted therapy. Patients often experience acute complications, including oral mucositis and reduced salivary secretion, depending on the type and extent of treatment. These complications affect essential functions such as speech, swallowing, hearing, and breathing, crucial to individual and social interaction [15,16]. Therefore, post-treatment management of HNC is vital, and it is essential to evaluate and improve PE regularly.

The PE evaluation of patients with HNC should be multi-dimensional. It must include various aspects that patients experience during the care process, such as emotional and social support and functional outcomes. Additionally, assessing patients’ needs throughout their treatment journey is essential to providing quality care [6].

However, the current evaluation items for patients with HNC centered on physical aspects such as functional disability and limiting factors experienced by patients. Psychological factors are as important as physical factors in the QoL of patients with HNC. Considering various aspects of PE is crucial in bringing about satisfaction with medical services, treatment adherence, and positive treatment outcomes [17]. To qualitatively address PE, it is necessary to have a comprehensive view and effectively utilize a framework that structurally evaluates the characteristics of each factor and the user’s perspective on the factors [18]. To the best of our knowledge, there needs to be a more comprehensive evaluation of various factors, such as the emotions experienced by patients, treatment tools, treatment information and resources, hospital environment, and hospital system.

Meanwhile, QoL has become a fundamental concept in healthcare as the life expectancy of patients with chronic disease increases with the advancement of healthcare treatment technology. As the patient lives longer, the quality of PE related to treatment becomes essential. Accordingly, methodologies that focus on the needs of patients’ non-physical attributes, such as a need-based value measurement, have received attention [19]. However, compared to the increasing importance of QoL measurement in healthcare, standardized evaluation tools are severely lacking. Most present evaluation items only in the passive range of disease or treatment [20]. Therefore, an integrated view and evaluation of patient needs related to PE and QoL from various aspects is essential to increase healthcare service satisfaction and treatment outcomes for patients with HNC who require long-term treatment and rehabilitation.

### 1.2. Study Aim and Research Questions

To improve the PE of patients with HNC, this study aimed to propose HNC PE evaluation items from the diverse aspects of patient QoL by conducting a qualitative comparative study. The specific research questions are as follows:

(1) What aspects of PE should be comprehensively considered to improve patients’ QoL?;
(2) What aspects of PE do current HNC measures address? Also, what aspects are lacking?;
(3) What are users’ needs related to HNC treatment, and what new PE evaluation items can be added through this?

## 2. Materials and Methods

**Figure 1:**
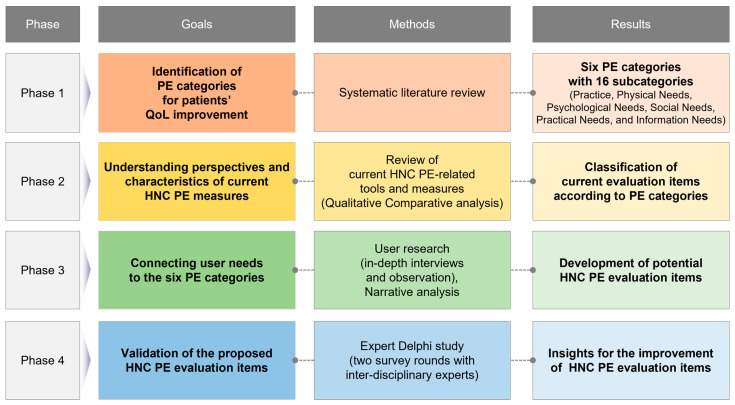
The research process.

According to the study purpose, the four-phased research process is as follows: Phase one was to identify comprehensive PE categories for patients’ QoL improvement. Through a systematic review, various aspects that affect the improvement of PE were analyzed, and integrated PE categories were derived. In the second phase, the perspectives and characteristics of the current HNC or cancer patients’ measures were reviewed by comparing the PE categories derived from the first phase. In the third phase, user research and narrative analysis were performed to identify and connect the user needs to the six PE categories and the current HNC PE measures. The potential HNC PE evaluation items were derived through a qualitative comparative analysis with results from phases one and two. Phase four was to verify the proposed evaluation items. A Delphi study was conducted with interdisciplinary experts to present the final HNC PE evaluation items and provide insight for improvement (Figure 1).

### 2.1. Phase 1: A Systematic Literature Review of PE Factors

In the first phase, a systematic literature review was conducted. In exploring the factors for improving PE, studies in four electronic databases (Google Scholar, Web of Science, PubMed, and Taylor & Francis) were searched using MeSH terms of patient-centered care, health-related quality of life, quality improvement, and keywords of patient experience and healthcare service design. The Taylor & Francis journal was selected because it covers a wide range of cross-disciplinary social science and medicine knowledge. It helps understand diverse aspects of research and practice in psycho-social, behavioral, and health systems.

The papers published between 2015 and 2021 were searched. As interest in PCC increases and knowledge accumulates, it has become essential to understand the updated PE factors based on recent research and actively reflect them in measures [21]. Therefore, starting in 2021, when the research began, the publication period was limited to after 2015 to prioritize the inclusion of recently published research papers on PE. The peer-reviewed articles, papers published in English, and empirical studies showing specific PE factors affecting PCC in healthcare services were included in the eligibility criteria.

Three researchers reviewed the data separately and compared the results with the others to confirm no errors. After the two researchers independently reviewed the abstracts and screened papers, the third researcher participated to reach a consensus. For the full-text review, the two researchers mainly conducted the review and selected final papers with the third researcher’s consensus on the selection.

The literature search identified 808 articles in the first round after reviewing abstracts using patient needs, barriers, evidence, improvement, protocols, requirements, or insights. We included papers focusing on healthcare, service design, PE, and communication for the inclusion criteria, and 135 papers were selected. After carefully reviewing the full text of the selected studies regarding whether the PE factors were explicitly addressed from the empirical studies, 24 papers were selected. Finally, 11 studies that directly included factor analysis to improve PE during treatment were chosen for final analysis.

The content analysis of the selected studies identified 55 general PE factors. The researchers used Microsoft Excel 2021 to collect and organize data for the analysis. The researchers identified the specific PE factors through the content analysis, and the factors were grouped into categories based on similar attributes. The two researchers were independently involved in the data collection and content analysis. After the initial data extraction, three researchers gathered and performed the thematic analysis to categorize the PE factors according to their attributes and aspects. This phase builds a framework for analyzing currently used evaluation tools derived from patient needs.

### 2.2. Phase 2: Analysis of Current QoL Evaluation Tools

In the second phase, we reviewed the current QoL evaluation measures to examine which items comprised the measurement for patients with HNC currently in use and which factors should be added. We included cancer patient measurements because the tools are generally used for the QoL evaluation of patients with HNC. Three generic and four specific scales were selected for the analysis. These were included as frequently used and validated tools to assess QoL satisfaction or PE in patients with cancer or HNC. Content and thematic analyses were used to determine the PE factors to which each item corresponded. The same three researchers participating in the literature review were also involved in this stage.

The QoL of patients with HNC must be measured multidimensionally, and a scale that covers a wide range of items is required [22]. QoL measures can be classified into two groups: generic scales that evaluate QoL and health status independently of all pathologies and specific scales that evaluate specific diseases, fields, symptoms, and treatment methods. Among these, (1) the Patient Satisfaction Questionnaire (PSQ-18) has been validated for flexible use in different settings [23]; (2) the 36-Item Short Form Survey (SF-36), which is an often-used measure tool for assessing patients’ quality of life [24]; and (3) the CAHPS developed for assessing adult cancer patients’ experiences among all cancer treatment settings [25]; were reviewed for generic scales.

We selected the four specific measures most frequently used to analyze the QoL of patients with HNC according to the International Quality of Life Conference held in Virginia, USA, in 2002: (1) Functional Assessment of Cancer Therapy Quality of Life Measurement System (FACT-H&N) [26], (2) University of Washington Quality of Life Questionnaire (UW-QOL) [27], (3) European Organization for Research and Treatment of Cancer Quality of Life Questionnaire Core 30 (EORTC QLQ-C30) [28], along with the European Organization for Research and Treatment of Cancer Quality of life-Head and Neck Cancer Module (EORTC QLQ-H&N35) [29], and (4) satisfaction with cancer information profile (SCIP) for patients with HNC.

### 2.3. Phase 3: Analysis of HNC Patient Needs

In the third phase, we conducted interviews and observations with patients, their caregivers, and medical staff to identify potential items for improving the PE evaluation for patients with HNC from multiple perspectives. The user research included a family-oriented care group that could represent their opinions and thoughts on behalf of older patients. We also had otolaryngology medical staff who indirectly represented a patient’s needs through close interactions with the patient during treatment. They were included in the user research group because they could help identify latent needs that patients did not address directly.

The interviews and observations were analyzed using data from 31 individuals collected between August and October 2021 (Table 1). We conducted nine one-hour interviews using a semi-structured questionnaire built by researchers through a literature review and five observations. The questionnaire received feedback from three otolaryngologists and went through a process of reviewing the appropriateness of items, clarity of content, and ethical issues. If the patient could not communicate on his/her own and the caregiver was a family member, the caregiver attended the interview together. If the patient could communicate on his/her own, the patient and caregiver attended individually. They freely answered questions while recalling memories of the order of the treatment journey that the patient had experienced thus far. The researcher also attended five medical consultations with the consent of the patients and caregivers for the observation. Researchers stayed behind the room, observing the users’ activities, behaviors, and interactions; the objects used; the information delivered; and the physical space environment.

Before proceeding with the user research, ethical approval was obtained from the hospital’s institutional review board. The otolaryngologist briefly introduced the purpose of the study and the contents of the questions to patients and caregivers right after the counseling session. When informed consent was obtained from the participants, the researchers conducted interviews and observations in an individual room. The participants were assured that the anonymity of the collected data was strictly guaranteed, and it was also explained that they could leave the session at any time if they wanted. Patients included all the various treatment itineraries before or after surgery without considering the subsite of HNC because the study goal was to identify the overall needs of patients during the treatment journey for HNC instead of focusing on a specific subsite.

The data collected from the user research were recorded and fully transcribed. The content analysis introduced by Graneheim and Lundman [30] was applied to identify patient needs according to the treatment journey. Based on their methodology, the interview and observation data derived meaning units that capture the core meanings of the contents corresponding to PE enablers and barriers. These were summarized as keyword-oriented core codes. The derived codes were compared with the previously identified PE factors from the literature review and matched to related items.

### 2.4. Phase 4: Suggestion of HNC PE Evaluation Items through Validation

In Phase 4, the potential items for patient-centered integrative HNC PE evaluation were developed by synthesizing the three materials identified in each phase: general PE factor categories (phase 1), existing HNC PE evaluation scales (phase 2), and the HNC patient’s needs (phase 3). The researchers conducted a combination of literature review, current measurement analysis, and user research to closely connect the analysis results derived at each stage and systematically structure the relationship between the results.

Evaluation criteria were set using the five items introduced by Tsang et al. [31] to validate the items derived through phases one to three. The researchers modified ‘comprehensiveness’ to ‘importance’ to convey the meaning more clearly, in addition to the four criteria of ‘clarity’, ‘diversity’, ‘availability’, and ‘ethics’ among the five criteria (Table 2).

The validation of the PE evaluation items derived in this study followed the Delphi method for expert consensus introduced by Steinmann et al. [32]. For validation, 10 experts, including HNC specialists, cancer/otolaryngology medical staff, healthcare service professionals and managers, and QI researchers, were purposively recruited (four females and six males). Those eligible for recruitment included medical staff or experts who were involved in carrying out this study or were familiar with or in charge of QoL assessment or the work of the QI team within the hospital. The Delphi survey was conducted twice in total. Each round validated PE evaluation items on a four-point Likert scale: (Very Important (4), Important (3), Moderately Important (2), or Not Important (1)) against five criteria shown in Table 2. After evaluating each factor on the Likert scale, if the respondent answered Moderately Important (2) or Not Important (1), they were asked to write the reason, suggestions, or related opinions in free text.

When analyzing the results, the average values of each respondent for each item were calculated. If more than 80% of experts (at least eight out of 10) gave an average value of 3 or more, the researchers retained the items. On the other hand, if more than 50% of the participants (at least five out of 10) showed an average value of less than 3 points, the items were excluded. The items excluded in the first round were treated as discordance and revised and supplemented for the second survey round. The second round was also conducted the same way as the first round, and the items for which more than 80% of the participants gave average scores of 3 or more were presented as the final evaluation items.

## 3. Results

The research consisted of four phases, each employing different methods to achieve its specific goal. In the first phase, the diverse aspects of PE factors were reviewed to identify comprehensive PE categories for evaluating a wide range of QoL patients. The second phase involved the review of the current HNC PE-related measures to identify the potential aspects to be improved. The third phase described the process of drawing user needs from the user research to suggest potential HNC PE evaluation items by qualitatively comparing the results of the three phases mentioned above. As mentioned, the literature revealed that different aspects of PE factors should be addressed holistically for the patient QoL. However, the current HNC PE-related measure covered only some aspects of PE but focused on the disease and treatment-specific subject. The researchers conducted the study phase by phase, presenting evidence-based justification for the previous aspects and systematically presenting the process of extracting specific and practical evaluation items.

### 3.1. Phase 1: Identification of Factors Influencing PE Improvement

After reviewing the literature, the selected 11 studies identified 55 factors affecting PE improvement. The researchers performed content analysis to classify the identified factors based on their similarity. The 55 factors were classified into six categories (*Practice* (*Pr*), *Physical Needs* (*PhN*), *Psychological Needs* (*PsN*), *Social Needs* (*SN*), *Practical Needs* (*PrN*), *and Information Needs* (*IN*)) and 16 subcategories (Phase 1 in Table 3 (A–F)).

### 3.2. Phase 2: Analysis of Current HNC PE Evaluation Tools

After examining the specific items to evaluate PE with HNC, 200 items were investigated, with 86 items identified on the generic scale and 114 items identified on the specific scale. The related items were classified according to the category of PE factors derived in Phase 1; duplicate items were excluded, and 33 items were identified (Phase 2 in Table 3 (A–F)).

Current HNC PE evaluation items were used in all subcategories except for [Pr2] skill, [Pr4] organization of *Pr*, and [IN2] education of *IN.*

Items related to reviews/discussions of prescription drugs were identified for [Pr1] coordination of *Pr*, and items related to reviews or discussions of prescription drugs were identified. The [Pr3] Care Plan presents sufficient discussion regarding treatment after the cancer surgery decision as an evaluation item. In the [Pr5] QI (quality improvement), attitudes such as encouraging the patient to ask questions when necessary and the medical staff to carefully listen and display respect/decency toward the patient’s words were suggested. In [Pr6], Management, the appropriateness of the timing, content, and quantity of information provided to patients, their caregivers, or their families was assessed. When this result was applied to the category of PE factors established in Phase 1, the following items were relatively lacking: (1) patient or caregiver’s active participation (collaboration and involvement) in the treatment process; (2) systematic information delivery method provided by medical staff and the hospital (dedicated room, written and verbal information, and scheduled team meetings); and (3) the organization’s leadership and vision for improving PE.

Regarding *PhN*, the specific items for identifying the pain and symptoms patients experience due to cancer or surgery were included, reflecting the physical aspects of patients with HNC.

*PsN* included pain caused by cancer or surgery, diagnosis of emotional problems (anxiety, depression, and worry), and related advice, help, and counseling. However, emotional support from family and social relationships has not yet been covered.

Regarding *SN*, [SN1] communication mainly evaluated the limitations and inconvenient aspects of explaining patients’ opinions during direct conversations or phone calls with medical staff, caregivers, or family members. Compared with the items presented in the general PE factors, items such as patient, caregiver, or family involvement in the treatment process, devices to promote communication, and attitude training of medical staff were excluded from the evaluation. [SN2] Support revealed the limitations of the scales, which only evaluated simple information delivery rather than the active involvement of patients and caregivers in setting up treatment plans. For [SN3], Respect, encouraging patients to file complaints was not mentioned. [SN4] Responsiveness evaluates whether the medical staff devotes sufficient time to patients. However, reception and response to patient complaints were not included. In summary, there needed to be more items to reflect patients’ voices, such as their active intervention and participation in the treatment process, the decision making of patients, caregivers, and families, and the reception of and responding to patients’ complaints.

Regarding *PrN*, evaluations were made concerning reservations for treatment, staff contact, result sharing, and noise control. However, items such as treatment costs and support, which are practically essential to patients; information on treatment after discharge; consideration for caregivers and family members; and information on complaints still need to be sufficiently evaluated.

In *IN*, [IN1], information and knowledge were configured to help patients make decisions by evaluating whether the information provided was sufficient for various contents. However, no separate evaluation items concerning [IN2] education were identified. Therefore, it was necessary to continuously check information, evaluate whether educational materials were provided, and verbally explain the information to patients.

### 3.3. Phase 3: Identification of Insights for Potential Items Based on Patients with HNC Needs

The matching of HNC patient needs to the corresponding PE factor category established in Phase 1 identified 31 PE evaluation items: two from *Pr*, three from *PhN*, three from *PsN*, 11 from *SN*, four from *PrN*, and eight from *IN* (Phase 3 in Table 3 (A–F)).

Two potential items appeared in *Pr*: consistent treatment and information delivery through effective communication between departments, and comprehensive guidance on the essential examination methods and procedures according to the treatment stage. This indicated that patients prefer comprehensive information about the entire journey rather than information about the treatment process in segments. In addition, obtaining consistent and efficiently linked treatment information between departments is essential, rather than listening to the conflicting opinions of each department that is visited for collaboration.

For *PhN*, patients who wanted to obtain positive results from surgery were identified to assess the treatment skills of medical staff; sufficient advance guidance on scars, complications, and external disorders that may continue to affect their appearance; and response and treatment provided by medical staff regarding various symptoms that they may experience after surgery.

In *PsN*, the medical staff’s attitude of fully emphasizing with and supporting the anxiety and worry experienced by patients and caregivers was found to be a critical evaluation factor.

For *SN*, patient-friendly communication tools and attitudes to build bonds with medical staff and guidance on support measures for treatment costs can be interpreted as essential for improving PE.

Regarding *PrN*, the patients’ representative needs were minimization of the time lag to receive surgery as quickly as possible, assistance with reservation procedures, provision of convenient transportation and accessibility to hospitals, and creation of a pleasant hospital environment.

Regarding *IN*, in addition to comprehensive information guidance on the entire treatment process, it has been confirmed that various types of information about the long-term journey before and after treatment, such as the cause of cancer, updates on postoperative progress, care plans and self-management methods after discharge, and access to reliable information, are required. In addition, beyond the simple provision of information, patients actively wanted to return to their daily lives and continue treatment through education and data distribution on rehabilitation, alcohol and smoking cessation, and other activities.

Table 3 (A–F) summarizes the study results identified from each phase (Phase 1: literature review; Phase 2: current PE scales analysis; and Phase 3: patients with HNC needs analysis) and explains the process of deriving the potential PE evaluation items by synthesizing the items of the three phases. Initially synthesized items were compared with existing HNC PE evaluation items to, finally, identify newly derived items. As a result, nine from *Pr*, three from *PhN*, three from *PsN*, 11 from *SN*, nine from *PrN*, and four from *IN* (total n = 39) were identified. Each derived item was assigned a code and indicated who, among patients (P), medical staff (M), and hospital administrators (H), should be asked questions.

### 3.4. Validation

Experts verified the 39 potential items through a Delphi study (two survey rounds). In the first evaluation round, among the 39 potential items, 17 received an average value of three or more from more than 80% of respondents. They were included as items for PE evaluation based on the consensus of experts. On the other hand, 11 items were excluded that received an average value of fewer than three points from more than 50% of respondents. The remaining 11 items that were not included or not excluded were disagreements between experts.

The 11 items that resulted in disagreement were discussed by researchers based on free-text comments written by experts, and the items were revised by clearly conveying the meaning and specifying the content. In addition, among the 11 excluded items, three items with high importance values and low clarity values compared to the average scores were judged to have high importance as evaluation items by experts. However, the meaning was not clearly conveyed, leading to disagreement. Thus, the three items were modified similarly to the discordance factors. For the 14 revised items, a second survey round was conducted, targeting the same experts who participated in the first round to evaluate the importance of each item once again as a PE evaluation item. The evaluation process and method was the same as in the first round.

As a result of the second survey, out of 14 items, eight items were included that received an average value of three or more by more than 80% of respondents. The three items were finally excluded because they received an average value of fewer than three points by more than 50% of respondents, and there was disagreement among respondents about the remaining three factors.

In summary, through two survey rounds, 25 out of 39 items were included, 11 were excluded, and the remaining three resulted in disagreement (Table 4). The 25 included items were revised again based on the experts’ free-text comments for clarity of meaning and content specification. Table 5 shows the items included in the HNC PE evaluation, alongside average scores and standard deviations.

## 4. Discussion

### 4.1. Summary

Thirty-nine items were presented in the three phases of exploring the HNC PE evaluation items (nine from *Pr*, three from *PhN*, three from *PsN*, 11 from *SN*, nine from *PrN*, and four from *IN*). To validate the items, a Delphi study consisting of two survey rounds was conducted to validate the items. After two survey rounds, 25 items were included, 11 were excluded, and three were disagreed with.

For the included items, six items (organizing regular team meetings to share patient information, establishing a medical environment for patients’ emotional support, delivering information in written and verbal forms, encouraging collaboration between patients and medical staff for treatment decision making, managing patient satisfaction data, and ensuring the patient’s participation in decision making) were identified in *Pr*. *PhN* involved three items: the medical staff’s adequate responses to complications, communication difficulties, and nutritional imbalances, as well as their provision of necessary information after surgery. *PsN* included one item: the medical staff’s adequate responses to patients’ emotional and cognitive condition. Regarding *SN*, seven items were identified: the medical staff providing alternative non-communication tools, using easy-to-understand medical terminology, delivering essential information, encouraging the maintenance of patients’ rehabilitation, ensuring patients’ participation in each stage of the treatment process, and the hospital providing a communication channel. *PrN* included five items: the medical staff’s comprehensive treatment information delivery, the patient complaint-filing procedure and allowing for contact with medical staff in case of emergency, the provision of alternative treatment options, and the support programs provided by the hospital for families and caregivers. IN involved: accurate and reliable information, comprehensive information packages, and patient education programs.

The 11 excluded items involved the medical staff’s time spent with patients, the discussion of PE results and improvement plan, the provision of advance notice of the medical staff’s rounding time/schedule, the availability of interpersonal skills training, the provision of access to medical services for socially vulnerable groups, the information being provided on support methods to reduce the financial burden on patients, the transportation and parking assistance, noise control efforts, and the patients’ access to information to receive updates on their condition after surgery. Most of these items were related to hospital systems and social policies, so there were limits to generalizing HNC PE evaluation items for patients. The experts who participated in the validation viewed that the excluded items could be limited to evaluating the patient’s treatment service satisfaction. However, measuring the need-based value for the patient’s QoL was difficult. Additionally, because financial support and transportation were not the items a specific hospital or individual medical staff could solve, most experts were skeptical about selecting these items as official evaluation indicators.

The three discorded items were empathy and the motivation of medical staff to support patients’ emotions and rehabilitation treatment, and the minimization of waiting time for surgery and studies. These items are susceptible to who was asked and where they worked. Therefore, different response results may be obtained depending on the respondent’s personal preferences and hospital environment. This study conducted a Delphi study targeting medical staff working at affiliated organizations of university hospitals that operate with the same vision and principles. However, personal tendencies could not be controlled. Therefore, in the future, additional research is needed to develop these discorded items into evaluation items by considering these issues. Thus, there were differing opinions on developing them into objective evaluation indicators.

Looking at the above results, *Pr* emphasized patient information data sharing and management and the establishment of tools and environments to increase patient participation in the decision-making process. *PhN* and *PsN* emphasized adequate responses to the patient’s physical discomfort, such as complications, communication, and nutrition, as well as emotional and cognitive aspects that may occur after surgery. For *SN*, providing various tools and methods to support seamless communication between patients and medical staff was emphasized. *PrN* highlighted the delivery of comprehensive treatment information and support programs for families and caregivers. In *IN*, quality aspects of information and patient education were highlighted.

### 4.2. Implications and Study Limitation

**Figure 2:**
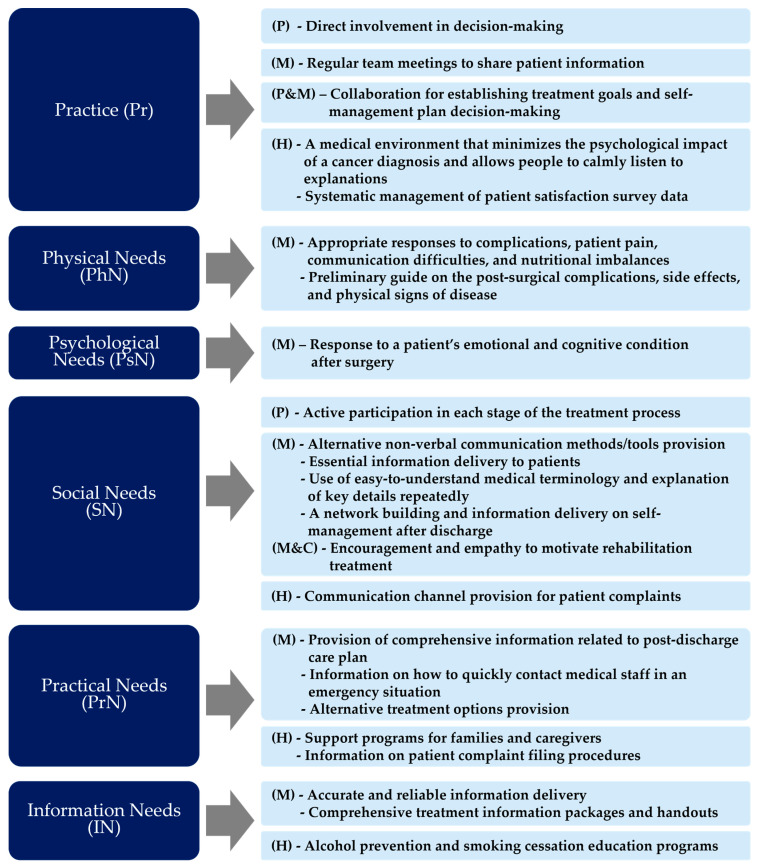
HNC PE evaluation items to be considered for achieving patient-centeredness. (P—patients, M—medical staff, H—hospital administrators, C—caregivers).

In this study, we explored the opportunity to improve HNC PE evaluation step by step by reviewing the literature, analyzing existing HNC PE evaluation scales, and identifying patient needs from user research. Through the three phases, various aspects of PE improvement were found to be critical for suggesting an integrative evaluation model. Figure 2 shows the HNC PE evaluation items to be considered for each category to ensure patient-centeredness. Each item was reclassified within a category based on the relevant stakeholders.

The evaluation scales that have been used in the current hospital settings are related to structural and outcome indicators that can tangibly capture patients’ opinions on evaluation by focusing on patient pain and symptoms, the hospital environment, and treatment management systems. In comparison, the suggested evaluation items in the study highlight the comprehensive and quality information delivery, the tools and environments to support patients’ participation and communication, patient data sharing and systematic management, the response to patients’ pain and emotion, and the support/education programs for diverse stakeholders. These results clearly show that the suggested items deal with various aspects of information (content and scope, communication tools, data management, and education) as necessary. Compared to the existing HNC evaluation items we looked at earlier, which are the lack of patient participation and systematic information delivery methods, the items presented in this study considered various aspects of information such as content, delivery method, management, and education to increase patient participation and the efficiency of information delivery.

The current evaluation items primarily encompass the patients’ functional outcomes and the structured, system-oriented complex factors for assessing HNC PE. In contrast, the newly formulated items, driven by patient needs, capture intangible and softer aspects. These include the tools and methods to enhance patient engagement and decision making during treatment. For instance, they provide specific information to facilitate interactions among stakeholders. This shows that the patient-centered perspective was strengthened by reflecting patients’ needs on the items.

Therefore, in this study, when compared with existing evaluation items, critical insights for HNC PE evaluation improvement were explored by integrating items that reflect patients’ needs and preferences. In addition to the functional aspects of the various services that patients experience during the treatment process, the researchers encompassed the methodological support and empathy of the medical staff, the way they deliver services, and the qualitative aspects of the contents.

However, as a limitation of this study, the literature review and user research targeted a limited number of people and literature. Additional and continuous literature research and interviews are required to verify the generalization and versatility of the evaluation items further developed in this study. In addition, the three items related to the medical staff’s attitude and the minimization of waiting time did not reach a consensus among experts in both surveys. Although these are essential indicators for improving PE when viewed individually, it appears to be subjective or due to differences in hospital environments and systems and difficult to evaluate as an objective indicator. Additionally, there are potential barriers to implementing the proposed model related to resource constraints, inadequate staff training, and technological infrastructure. Additional discussion is needed on essential items such as discussing PE results and training medical staff to better communicate with patients. These items may be judged to have varying importance depending on the user’s perspective. They may be applied differently in each clinical setting because the way hospitals are operated and socio-cultural needs differs between countries and cultures. Therefore, further studies should be conducted to additionally review these potential barriers and challenges and to suggest measures for practical application of the proposed model according to distinct healthcare settings.

## 5. Conclusions

We explored an opportunity to improve patient-centered integrative evaluation for HNC PE by synthesizing literature review data, the HNC evaluation measures currently used, and the practical needs of patients with HNC. Due to long-term treatment, frequent consultations, and complications, patients with HNC experience decreased overall life satisfaction. Thus, to improve successful treatment and QoL, it is necessary to consider various experiential factors from diverse user perspectives and to link them to an integrative evaluation system.

In this study, we structurally identified the insights for the PE evaluation improvement that reflect the importance of patient-centered perspectives and integrative PE management. For improving HNC PE, we adopted various aspects, such as pain and psychology of patients; efforts and skills of medical staff; interaction and intervention of patients, caregivers, and medical staff; hospital system operation; treatment plan; rehabilitation after discharge; treatment cost; provision and management of treatment information; and education, among others. We introduced a three-phased approach to systematically analyze these various aspects and present the data classification and derivation process as objectively as possible. We synthesized the results by applying them to a literature-based, objective framework. We conducted two Delphi studies with 10 experts to verify the evaluation items presented through a qualitative study. However, for the items presented in this study to be used practically in real-world clinical settings, further validation studies or pilot testing would be necessary to gauge the effectiveness and reliability of the proposed evaluation model. Additionally, the research scope did not cover all aspects of PE factors for QoL of patients with HNC. Thus, more in-depth research is still needed to ensure the research findings include a wide range of potential aspects to be widely utilized.

As the patient-centered integrative HNC evaluation tool extracted potential items based on the actual needs of patients with HNC, they are expected to be utilized to improve the PE of patients with HNC in actual hospital settings. In addition, rather than presenting specific evaluation items, conceptual sentences with core meanings were suggested so that the specifications of the items could be flexibly adjusted in each setting. The study results can be used as guidelines for improving, correcting, or supplementing current evaluation tools in each field. These results serve as a basic framework for developing hospital QI evaluation materials to improve PE satisfaction among patients with HNC. Hospitals can flexibly select and apply items according to their situations and environments and can measure tangible user experience satisfaction through quantitative scores such as the Likert Scale.

## Figures and Tables

**Table 1:** The participants involved in the user research.

User Research Method	Users Involved (Gender, Age)	Date
In-depth Interview	Resident (female, 31)	27 August 2021
	Specialist (male, 45)	10 September 2021
	Resident (female, 31)	10 September 2021
	Patient (male, 65)/Caregiver (female, 39)	27 August 2021
	Patient (male, 56)/Caregiver (female, 54)/Specialist (male, 45)	27 August 2021
	Patient (male, 70)/Caregiver (female, 68)	27 August 2021
	Patient (male, 56)/Caregiver (female, 60)/Specialist (male, 45)	1 October 2021
	Patient (male, 70)/Caregiver (female, 68)	7 October 2021
	Patient (female, 74)/Specialist (male, 45)	15 October 2021
Observation	Patient (male, 65)/Caregiver (male, 39)/Specialist (male, 45)	27 August 2021
	Patient (male, 48)/Caregiver (male, 49)/Specialist (male, 45)	27 August 2021
	Patient (male, 70)/Caregiver (female, 68)/Resident (female, 31)	10 September 2021
	Patient (male, 56)/Caregiver (female, 60)/Specialist (male, 45)	5 October 2021
	Patient (female, 74)/Specialist (male, 45)	15 October 2021

**Table 2:** The assessment questions for validation.

No.	Criteria	Items for Validation
1	Clarity of meaning	The factors described for each category were straightforward to understand
2	Diversity of stakeholder perspectives	The resulting factors covered various aspects of PE categories
3	Availability of use in hospital settings	The suggested factors can be used to evaluate QoL of HNC patients in the future
4	Importance as an evaluation indicator	The identified factors can be considered important regarding the QoL of HNC patients
5	Ethics that does not infringe on individual privacy	Some of the factors violate privacy issues

**Table 3:** The process of deriving potential HNC PE evaluation items: (**A**) ***Category of Practice*** (**B**) **Category of *Physical Needs***. (**C**) **Category of *Psychological Needs***. (**D**) **Category of *Social Needs***. (**E**) **Category of *Practical Needs***. (**F**) **Category of *Information Needs*** (Stakeholders ^†^—P: patients, M: medical staff, H: hospital administrators).

(**A**)					
**Subcategory**	**Phase 1**	**Phase 2**	**Phase 3**	**Initial Synthesis ** **(*Stakeholders*) ^†^**	**Newly Derived Items** **(*Stakeholders*) ^†^**
[Pr1] coordination	[a] scheduled team meetings [b] structured team communications focused on individual patient care	[c] review and discussion of prescription drugs	[d] consistent treatment and information delivery through effective communication between departments	- regular team meetings to share patient information (a, d) (*M*) - regular team communication about patient medication prescription and care (b, c) (*M*)	[Pr1-1] regular team meetings to share patient information (a, d) (*M*)
[Pr2] skill	[a] reduce repetitive assessments by multiple staff [b] a dedicated space for diagnosis [c] written and verbal discharge information to patients	-	[d] comprehensive guidance on essential examination methods and procedures according to the treatment stage	- the number of evaluations between employees (a) (*M*) - a dedicated space for cancer diagnosis (b) (*M*, *P*) - the treatment information provided in written and verbal forms (c, d) (*M*, *P*)	[Pr2-1] a dedicated space for cancer diagnosis (b) (*M*, *P*) [Pr2-2] the treatment information provided in written and verbal forms (c, d) (*M*, *P*)
[Pr3] care plan	[a] the practice’s collaboration with the patient/caregivers for treatment goals and self-management planning [b] the practice’s coordination with healthcare providers/specialists/consultants for pertinent demographic and clinical data	[c] adequate discussion of treatment after cancer surgery decision	-	- collaboration of patients/medical staff/caregivers on treatment goals and self-management plans (a, c) (*M*, *P*) - patient demographics and clinical data shared among medical staff (b) (*M*)	[Pr3-1] patients/medical staff/caregivers collaborating on treatment goals and self-management plans (a, c) (*M*, *P*)
[Pr4] organization	[a] reliable complaints data sets to govern care quality, safety, and patient centricity [b] leadership committed and engaged to unify and sustain the organization in a joint mission [c] a vision clearly and constantly communicated to every member of the organization	-	-	- patient complaint data systematically managed to ensure quality care, safety, and patient-centeredness (a) (*H*) - appropriate leadership to integrate and maintain the organization (b) (*M*) - the vision communicated to every organization member clearly and consistently (c) (*M*)	[Pr4-1] patient complaint data being systematically managed to ensure quality care, safety, and patient-centeredness (a) (*H*)
[Pr5] QI	[a] the opportunity to ask questions about recommended treatment [b] the doctors’ or nurses’ time spent with patients [c] patient involvement in decisions about their care [d] the practice’s ability to set goals, analyze, and act to improve performance on immunization rates, preventive care measures, resource use, and care coordination measures [e] structure and support provided by the organization for identifying and implementing QI [f] monitoring QI as part of staff performance assessment	[g] encouragement of medical staff to ask questions after cancer surgery decisions, before and after visits, if necessary [h] careful listening to and showing of respect and courtesy to the patient	-	- the patient being allowed to ask questions about the treatment method suggested by the doctor (a, g) (*M*, *P*) - the time per day doctors/nurses spend with patients (face-to-face interaction) (b) (*M*, *P*) - the patient being directly involved in treatment decision making (e) (*M*, *P*) - practice plan; analysis of the implementation and rates of immunization, preventive care, resource use, and care coordination measures (d) (*H*) - the hospital organization structure and support system adequate for QI analysis and implementation (e) (*H*) - QI being monitored during staff performance assessment (f) (*H*) - doctors listening to their patients and respecting them with courtesy (h) (*M*)	[Pr5-1] the time per day doctors/nurses spend with patients (face-to-face interaction) (b) (*M*, *P*) [Pr5-2] the patient being directly involved in treatment decision-making (e) (*M*, *P*) [Pr5-3] practice plan; analysis of the implementation and rates of immunization, preventive care, resource use, and care coordination measures adequate for QI analysis and implementation (d) (*H*)
[Pr6] management	[a] the practice’s ability to proactively identify patient populations for periodic care needs [b] collaboration and team management [c] monitoring the impact of specific interventions and change strategies [d] scheduling meetings to discuss PE results, plan improvements, and triangulating multiple data sources to understand feedback	[e] assessment of the adequacy of the details of the information provided to the patient, timing of information provided, amount of verbally transmitted information, amount of written information provided, information provided to family/caregivers, and information provided to the patient	-	- the patient population requiring periodic care proactively identified in advance (a) (*M*) - good collaboration between the teams (b) (*M*) - the information being well communicated to the patient/caregiver in detail; the information being delivered at the right time; the adequate amount of information being provided verbally and in writing (c, e) (*P*) - discussion of PE results and improvement of plans; verification of data (d) (*M*, *H*)	[Pr6-1] discussion of PE results and improvement plans and verification of data between the teams (b, d) (*M*, *H*)
(**B**)					
**Subcategory**	**Phase 1**	**Phase 2**	**Phase 3**	**Initial Synthesis ** **(*Stakeholders*) ^†^**	**Newly Derived Factors** **(*Stakeholders*) ^†^**
[PhN1] physical supports	[a] physical functioning, role functioning, arm symptoms, body image, and pain [b] physical comfort, freedom from pain, cognitive symptoms, and fatigue [c] treatment-related side effects; fertility concerns [d] physical manifestations of the disease (nausea and fatigue)	[e] pain, soreness, swallowing (mouth, chin, neck, shoulders) [f] swallowing food (liquids, solid foods, pureed formula) [g] discomfort (teeth, tooth loss, mouth opening, smell, taste, cough, hoarseness, appearance, eating (chewing), arm movement, itchy and dry skin, dry mouth, and sticky saliva) [h] pain due to cancer or cancer surgery [i] counseling on pain due to cancer or cancer surgery	[j] medical staff’s treatment method to minimize side effects [k] preliminary guidance on the possibility of external disorders and side effects after surgery [l] response and treatment of physical symptoms (communication difficulties, nutritional imbalance) that may occur during the recovery process after surgery	[PhN1-1] the pain level, symptoms, and dysfunction (physical functioning, role functioning, arm symptoms, body image, pain) (a, e, f, g, h) (*P*) - response/treatment appropriately performed to minimize side effects and relieve pain (b, j) (*P*) - side effects from treatment (c) (*P*) - physical signs and extent of illness (d) (*P*) - sufficient counseling for pain caused by cancer or surgery (i) (*P*) - preliminary guidance on the possibility of external disorders and complications after surgery (k) (*P*) - the medical team appropriately responding to and treating possible communication difficulties and nutritional imbalances during the recovery period after surgery (l) (*P*)	[PhN1-1] response/treatment being appropriately performed to minimize side effects and relieve pain (b, j) (*P*) [PhN1-2] preliminary guidance on the possibility of external disorders, complications after surgery, side effects from treatment, and physical signs of illness (c, d, k) (*P*) [PhN1-3] the medical team appropriately responding to and treating possible communication difficulties and nutritional imbalances during the recovery period after surgery (l) (*P*)
(**C**)					
**Subcategory**	**Phase 1**	**Phase 2**	**Phase 3**	**Initial Synthesis ** **(*Stakeholders*) ^†^**	**Newly Derived Factors** **(*Stakeholders*) ^†^**
[PsN1] Psychological support	[a] emotion, concentration, remembering, and fatigue [b] introduction of hourly proactive nursing rounds/weekly senior executive rounds [c] telephone contact to nurses regarding health concerns and clinical leads to review care information flow [d] ability to cope [e] social/family relationships [f] reassurance [g] emotional health	[h] pain advice and help [i] counseling for emotional problems such as anxiety or depression related to cancer or cancer surgery [j] suffering from emotional problems such as anxiety or depression [k] advice and help with emotional problems [l] feeling physically less attractive because of an illness or treatment [m] worries about cancer, anxiety, and retaining a hopeful attitude toward treatment	[n] medical staff’s empathy for the psychological aspects of patients/caregivers [o] emotional support from medical staff to relieve patients’ anxiety and motivate them to recover [p] appropriate response to the anxiety felt by patients/caregivers	- the patient’s emotional health index of concentration, remembering, fatigue, and loss of confidence (a, g, l) (*P*) - the nurse’s round time/schedule informed to the patient (b) (*M*, *P*) - the patient contacting a nurse over the phone about health concerns; making a phone call to the clinical lead to review care information (c, h) (*P*) - the patient maintaining a positive attitude toward treatment to overcome cancer (d, m) (*M*, *P*) - the patient maintaining relationships with family and acquaintances for psychological stability (e) (*P*) - support so the patient can receive treatment with peace of mind (f) (*M*, *P*) - advice about the patient’s pain and help to alleviate it (h) (*M*, *P*) - appropriate responses and counseling for emotional problems such as anxiety or depression (i, j, k, m, p) (*M*, *P*) - empathy and motivation among medical staff for the psychological aspects of patients/caregivers (n, o) (*M*, *P*)	[PsN1-1] the patient’s emotional health index of concentration, remembering, fatigue, and loss of confidence (a, g, l) (*P*) [PsN1-2] information regarding the nurse’s round time/schedule being provided to the patient (b) (*M*, *P*) [PsN1-3] empathy and motivation among medical staff for the psychological aspects of patients/caregivers (n, o) (*M*, *P*)
**(D)**					
**Subcategory**	**Phase 1**	**Phase 2**	**Phase 3**	**Initial Synthesis ** **(*Stakeholders*) ^†^**	**Newly Derived Factors** **(*Stakeholders*) ^†^**
[SN1] Communication	[a] family life; social encounters [b] involvement of patients and families at multiple levels in the care process [c] communication with caregivers [d] use of slogans and acronyms to promote communication [e] interpersonal skills training for staff [f] setting of behavioral standards for staff [g] use of filmed PE interviews as communication education	[h] problems with communication with medical staff, caregivers, and family members [i] inconvenience of phone calls [j] limitations of clarification	[k] use of terms that are easy for elderly patients to understand [l] sufficient and repetitive explanations to help elderly patients understand [m] various communication methods and tools in preparation for cases when verbal communication is difficult	- maintaining family life and social encounters during treatment (a) (*P*) - the patient and family being involved in the care process at multiple levels (b) (*M*, *P*) - the patients, caregivers, family, and medical staff communicating seamlessly during care (c, h) (*M*, *P*) - the slogans and acronyms used to promote communication (d) (*M*, *P*) - the interpersonal skill training conducted for staff; the behavioral standards for staff presented (e, f, g) (*M*, *H*) - the necessary information delivered to the patient; contacting the patient by phone if necessary (i, j) (*P*) - the terms used that are easy for older patients to understand; explanations being given several times to aid understanding (k, l) (*M*, *P*) - various communication tools provided in case verbal communication is not possible (m) (*M*, *P*)	[SN1-1] the various communication tools provided in case verbal communication is not possible (m) (*M*, *P*) [SN1-2] the necessary information delivered to the patient; and contacting the patient by phone if necessary (i, j) (*P*) [SN1-3] the terms used that are easy for older patients to understand; explanations given several times to aid understanding (k, l) (*M*, *P*) [SN1-4] the patient and family being involved in the care process at multiple levels (b, c, h) (*M*, *P*) [SN1-5] the interpersonal skill training conducted for staff; the behavioral standards for staff presented (e, f, g) (*M*, *H*)
[SN2] support	[a] the practice’s collaboration with the patient/caregivers for treatment goals and self-management planning [b] network support to readjust to living independently post-discharge [c] accessibility improvement for commonly excluded patient groups (complainants burdened by health conditions or language barriers)	[d] provision of service information for home care after discharge [e] interpretation service for foreigners [f] provision of information on patient support groups for patients and caregivers	[g] preliminary information on self-management, such as regular exercise and lifestyle to follow surgery [h] encouragement and consolation of medical staff to motivate rehabilitation treatment [i] comfort and encouragement from people around the patient [j] relief of the burden of treatment costs, such as additional non-coverage costs in case of emergency (medical cost support plan guide)	- medical staff collaborating with the patient/caregivers on treatment goals and self-management planning (a) (*M*, *P*) - the practice establishing a network and providing information to support self-management after discharge (b, d, g) (*M*, *P*) - accessibility being improved for commonly excluded patient groups (c, e, f) (*H*, *P*) - the medical staff and people around them providing encouragement and comfort to motivate rehabilitation treatment (h, i) (*M*, *P*) - plans or support being made to reduce the burden of treatment costs (j) (*H*, *P*)	[SN2-1] the practice establishing a network and providing information to support self-management after discharge (b, d, g) (*M*, *P*) [SN2-2] accessibility being improved for commonly excluded patient groups (c, e, f) (*H*, *P*) [SN2-3] the medical staff and people around them providing encouragement and comfort to motivate rehabilitation treatment (h, i) (*M*, *P*) [SN2-4] plans or support being made to reduce the burden of treatment costs (j) (*H*, *P*)
[SN3] respect	[a] staff encouragement of complaint procedures [b] explaining things to understand [c] listening carefully to patients [d] showing respect to patients for their needs and preferences	[e] easy-to-understand explanation (how easy it is to understand the information) [f] listening carefully and respectfully	[g] communication and efforts of medical staff to build bond and trust between patient and medical staff [h] maintaining a positive attitude based on objective facts rather than excessive hope [i] use of terms that are easy for patients to understand [j] a communication window where patients can express their feelings to medical staff	- communication channels being provided for patients and caregivers to file complaints (a, j) (*H*, *P*) - the doctor explaining the care information easily (b, e, i) (*P*) - the medical staff listening to the patient carefully (c, f) (*P*) - the patient’s needs and preferences being respected (d) (*P*) - efforts to communicate to build bonds and trust between patients and medical staff (g) (*M*, *P*) - the medical team maintaining a positive attitude (h) (*M*, *P*)	[SN3-1] communication channels being provided for patients and caregivers to file complaints (a, j) (*H*, *P*) [SN3-2] the medical team maintaining a positive attitude (h) (*M*, *P*)
[SN4] responsive-ness	[a] comprehensive and bespoke responding to improve complainant satisfaction [b] spending enough time with patients	[c] medical staff devote enough time to patients	-	- comprehensive and bespoke responses to increase customer satisfaction (a) (*H*, *P*) - the medical staff spending enough time responding to the patient’s needs (b, c) (*M*, *P*)	
(**E**)					
**Subcategory**	**Phase 1**	**Phase 2**	**Phase 3**	**Initial Synthesis ** **(*Stakeholders*) ^†^**	**Final Factors** **(*Stakeholders*) ^†^**
[PrN1] Access to care	[a] assistance in affording and access to care services not covered by insurance or transportation [b] health insurance coverage [c] assistance with transportation to appointments driving, offering refreshments, navigation through the center, and parking [d] finance, parking cost, and information about post-discharge care [e] medical staff available when needing care [f] process for providing appointments and alternative types of clinical encounters [g] easy access to information that outlines procedures involved	[h] quick and convenient booking process [i] calling immediately if specific symptoms or side effects occur [j] instructions on how to contact us after regular office hours [k] sharing cancer diagnosis results [l] ordering blood tests, X-rays, and other tests [m] follow-up for the explanation of test results	[n] minimization of wait times for surgery and examinations [o] staff help with guidance and reservation process [p] the location of the hospital closest to the patient’s home (accessibility utilizing transportation)	- assistance with health insurance coverage, finance, and parking costs (a, b, d) (*H*, *P*) - assistance with transportation to appointments driving, offering refreshments, navigation through the center, and parking (c) (*H*, *P*) - information provided about post-discharge care and procedures involved (d, g, k, l, m) (*M*, *P*) - medical staff being available when the patients need care (e, i, j) (*P*) - the doctor offering alternative types of clinical options (f, h, o) (*M*, *P*) - minimization of wait times for surgery and examinations (n) (*H*, *P*) - the hospital being close to home or in an easy-to-reach location; provision of convenient means of transportation (p) (*H*, *P*)	[PrN1-1] assistance with health insurance coverage, finance, and parking costs (a, b, d) (*H*, *P*) [PrN1-2] assistance with transportation to appointments driving, offering refreshments, navigation through the center, and parking (c) (*H*, *P*) [PrN1-3] information provided about post-discharge care and procedures involved (d, g, k, l, m) (*M*, *P*) [PrN1-4] medical staff being available when the patients need care (e, i, j) (*P*) [PrN1-5] the doctor offering alternative types of clinical options (f, h, o) (*M*, *P*) [PrN1-6] minimization of wait times for surgery and examinations (n) (*H*, *P*)
[PrN2] environment	[a] care for the caregivers through a supportive work environment [b] floor coverings to reduce noise [c] creation of family rooms [d] quiet spaces [e] signposting to complaint procedures to prevent patients from filing a complaint	[f] noise management for conversation	[g] pleasant hospital environment	- family rooms and care for the caregivers being provided through a supportive work environment (a, c) (*H*, *P*) - reduction of noise and provision of a quiet space (b, d, f) (*H*, *P*) - signposting of complaint procedures to prevent patients from filing a complaint (e) (*H*, *P*) - provision of a pleasant hospital environment (g) (*P*)	[PrN2-1] family rooms and care for the caregivers being provided through a supportive work environment (a, c) (*H*, *P*) [PrN2-2] reduction of noise and provision of a quiet space (b, d, f) (*H*, *P*) [PrN2-3] signposting of complaint procedures to prevent patients from filing a complaint (e) (*H*, *P*)
(**F**)					
**Subcategory**	**Phase 1**	**Phase 2**	**Phase 3**	**Initial Synthesis ** **(*Stakeholders*) ^†^**	**Newly Derived Factors** **(*Stakeholders*) ^†^**
[IN1] Information & knowledge	[a] more information for being involved, decision-making, or planning future care [b] access to individual information	[c] provision of sufficient information (whether there is a conflict of medications due to treatment; symptoms that can be felt after treatment; effect of treatment on workability, financial support, and future additional treatment; effect of treatment on appearance; expected time to recover; treatment impact on life; information on patient support organizations)	[d] prompt and continuous updates on postoperative progress [e] comprehensive information about the entire treatment process [f] delivery of additional information, such as the cause of cancer [g] information on care plans after discharge, self-management methods, and precautions [h] acquiring accurate and reliable information about HNC	- sufficient information being provided for decision making on future care (a, c, e, f, g) (*M*, *P*) - access to individual information for prompt and continuous updates on postoperative progress (b, d) (*M*, *P*) - acquisition of accurate and reliable information about HNC (h) (*P*)	[IN1-1] access to individual information for prompt and continuous updates on postoperative progress (b, d) (*M*, *P*) [IN1-2] acquisition of accurate and reliable information about HNC (h) (*P*)
[IN2] education	[a] information pack and handouts on treatment options, care navigation, and discharge processes to patients/families	-	[b] educational materials that can systematically and continuously continue rehabilitation training after treatment [c] preventive drinking and smoking cessation education program [d] provision of educational methods and materials for self-management after discharge	- information packages and handouts provided on treatment options, discharge procedures, and rehabilitation (a, b, d) (*M*, *P*) - preventive drinking and smoking cessation education programs provided (c) (*H*, *P*)	[IN2-1] information packages and handouts provided on treatment options, discharge procedures, and rehabilitation (a, b, d) (*M*, *P*) [IN2-2] preventive drinking and smoking cessation education programs provided (c) (*H*, *P*)

**Table 4:** Results of the survey rounds.

Category	Round 1 (n = 39)	Between the Rounds	Round 2 (n = 14)
Included	**17**	-	-
Excluded	11	3 modified	2 Included
			1 Excluded
		8 Excluded	-
Discordance	11	11 modified	6 Included
			2 Excluded
			3 Discordance
**Summary**	25 Included (17 from Round 1, 8 from Round 2) 11 Excluded (8 from Round 1, 3 from Round 2) 3 Discordance (Round 2)		

**Table 5:** The Included items for the PE evaluation of patients with HNC.

No.	Category	Subcategory	PE Factor (Item)	Average Score	Standard Deviation
1	Practice	Coordination	Organizing regular team meetings to share patient information	3.45	0.56
2		Skill	Establishing a medical environment that minimizes the psychological impact of a cancer diagnosis and allows people to calmly listen to explanations	3.20	0.40
3			Providing treatment information to patients through written information in addition to verbal explanations	3.68	0.49
4		Care Plan	Patients, medical staff, and caregivers working together to establish treatment goals and make decisions about self-management plans	3.55	0.52
5		Organization	Systematic management of patient satisfaction survey (feedback) data to increase patients’ treatment satisfaction (patient experience)	3.40	0.39
6		QI	Patients directly participating in decision making about treatment methods, including surgery, after a cancer diagnosis	3.45	0.57
7	Physical Needs	Physical Supports	Medical staff providing appropriate response and treatment to minimize complications that may occur in patients after surgery and to relieve pain	3.45	0.40
8			Advancing the availability of information on post-surgical complications, side effects from treatment, and physical signs of disease	3.18	0.53
9			Medical staff appropriately responding to and treating communication difficulties and nutritional imbalances that may occur during the recovery period after surgery	3.65	0.45
10	Psychological Needs	Psychological Supports	Diagnosing and responding to the patient’s mental (emotional and cognitive) condition after surgery, including concentration, memory, fatigue, and loss of confidence	3.48	0.44
11	Social Needs	Communication	If a patient is unable to communicate verbally due to a tracheostomy after surgery, providing alternative non-verbal communication methods and tools	3.4	0.64
12			Medical staff delivering/guiding essential information to patients	3.23	0.56
13			Using easy-to-understand medical terminology that is easy for older patients to understand; explaining key details repeatedly to help patients and their caregivers understand	3.55	0.53
14			Giving consideration to patients and their families to participate as much as possible in each stage of the treatment process (throughout the entire treatment process)	3.40	0.52
15		Support	Building a network and providing related information to support self-management after discharge	3.40	0.53
16			The medical staff and people around the patient providing encouragement and comfort to maintain and motivate rehabilitation training/treatment to restore the patient’s function	3.45	0.60
17		Respect	Providing a communication channel through which patients and caregivers can file complaints	3.40	0.74
18	Practical Needs	Access to Care	Providing comprehensive information on outpatient treatment, rehabilitation treatment, and self-management after discharge at the time of patient discharge	3.33	0.79
19			Providing guidance on how to quickly contact medical staff in the event of unexpected symptoms or complications	3.33	0.49
20			Providing alternative treatment options that patients can choose from in addition to the treatment methods suggested to them	3.08	0.51
21		Environment	Providing support programs for families and caregivers	3.08	0.69
22			Providing information on patient complaint-filing procedures	3.13	0.64
23	Informational Needs	Information and Knowledge	Obtaining accurate and reliable information about head and neck cancer	3.15	0.63
24		Education	Providing a comprehensive information package and handouts regarding treatment options, discharge procedures, and rehabilitation treatment	3.45	0.50
25			Providing alcohol prevention and smoking cessation education programs	3.23	0.61

## Data Availability

The data that support the findings of this study are available on request from the corresponding author. The data are not publicly available due to privacy or ethical restrictions.
